# Novel ELISA Protocol Links Pre-Existing SARS-CoV-2 Reactive Antibodies With Endemic Coronavirus Immunity and Age and Reveals Improved Serologic Identification of Acute COVID-19 *via* Multi-Parameter Detection

**DOI:** 10.3389/fimmu.2021.614676

**Published:** 2021-04-09

**Authors:** Rachel R. Yuen, Dylan Steiner, Riley M.F. Pihl, Elizabeth Chavez, Alex Olson, Erika L. Smith, Lillia A. Baird, Filiz Korkmaz, Patricia Urick, Manish Sagar, Jacob L. Berrigan, Suryaram Gummuluru, Ronald B. Corley, Karen Quillen, Anna C. Belkina, Gustavo Mostoslavsky, Ian R. Rifkin, Yachana Kataria, Amedeo J. Cappione, Wenda Gao, Nina H. Lin, Nahid Bhadelia, Jennifer E. Snyder-Cappione

**Affiliations:** ^1^ Department of Microbiology, Boston University School of Medicine, Boston, MA, United States; ^2^ Department of Medicine, Boston University School of Medicine, Boston, MA, United States; ^3^ PiBS Program, Boston University School of Medicine, Boston, MA, United States; ^4^ Section of Infectious Diseases, Department of Medicine, Boston Medical Center, Boston, MA, United States; ^5^ National Emerging Infectious Diseases Laboratories (NEIDL), Boston University, Boston, MA, United States; ^6^ Flow Cytometry Core Facility, Boston University School of Medicine, Boston, MA, United States; ^7^ Department of Pathology and Laboratory Medicine, Boston University School of Medicine, Boston, MA, United States; ^8^ Center for Regenerative Medicine, Boston University School of Medicine, Boston, MA, United States; ^9^ Renal Section, Department of Medicine, Boston University School of Medicine, Boston, MA, United States; ^10^ Renal Section, Department of Medicine, VA Boston Healthcare System, Boston, MA, United States; ^11^ MilliporeSigma, Burlington, MA, United States; ^12^ Antagen Pharmaceuticals, Boston, MA, United States

**Keywords:** SARS-CoV-2, COVID-19, antibodies, serology, nucleocapsid (N), receptor binding domain (RBD), ELISA

## Abstract

The COVID-19 pandemic has drastically impacted work, economy, and way of life. Sensitive measurement of SARS-CoV-2 specific antibodies would provide new insight into pre-existing immunity, virus transmission dynamics, and the nuances of SARS-CoV-2 pathogenesis. To date, existing SARS-CoV-2 serology tests have limited utility due to insufficient reliable detection of antibody levels lower than what is typically present after several days of symptoms. To measure lower quantities of SARS-CoV-2 IgM, IgG, and IgA with higher resolution than existing assays, we developed a new ELISA protocol with a distinct plate washing procedure and timed plate development *via* use of a standard curve. Very low optical densities from samples added to buffer coated wells at as low as a 1:5 dilution are reported using this ‘BU ELISA’ method. Use of this method revealed circulating SARS-CoV-2 receptor binding domain (RBD) and nucleocapsid protein (N) reactive antibodies (IgG, IgM, and/or IgA) in 44 and 100 percent of pre-pandemic subjects, respectively, and the magnitude of these antibodies tracked with antibody levels of analogous viral proteins from endemic coronavirus (eCoV) strains. The disease status (HIV, SLE) of unexposed subjects was not linked with SARS-CoV-2 reactive antibody levels; however, quantities were significantly lower in subjects over 70 years of age compared with younger counterparts. Also, we measured SARS-CoV-2 RBD- and N- specific IgM, IgG, and IgA antibodies from 29 SARS-CoV-2 infected individuals at varying disease states, including 10 acute COVID-19 hospitalized subjects with negative serology results by the EUA approved Abbott IgG chemiluminescent microparticle immunoassay. Measurements of SARS-CoV-2 RBD- and N- specific IgM, IgG, IgA levels measured by the BU ELISA revealed higher signal from 9 of the 10 Abbott test negative COVID-19 subjects than all pre-pandemic samples for at least one antibody specificity/isotype, implicating improved serologic identification of SARS-CoV-2 infection *via* multi-parameter, high sensitive antibody detection. We propose that this improved ELISA protocol, which is straightforward to perform, low cost, and uses readily available commercial reagents, is a useful tool to elucidate new information about SARS-CoV-2 infection and immunity and has promising implications for improved detection of all analytes measurable by this platform.

## Introduction

From the first reported case of COVID-19 caused by the virus SARS-CoV-2 in December 2019 (1, 2) there have been more than 127 million reported cases and 2.79 million deaths worldwide as of March 29, 2021. Common symptoms of SARS-CoV-2 infection include fever, cough, myalgia, and fatigue and these symptoms vary widely in magnitude, nature, and duration between individuals for reasons that are not clear to date (3, 4), with some individuals with confirmed infections remaining asymptomatic (5). Epidemiological evidence indicates silent viral spread *via* asymptomatic individuals within communities and the extent of this form of transmission is currently unclear (6). SARS-CoV-2 has homology to other alpha and beta ‘common cold’ endemic coronaviruses (eCoVs) in circulation, and cross-reactive T cell immunity to SARS-CoV-2 spike (S) and nucleocapsid (N) proteins are present in a substantial percentage of unexposed individuals (7–10). Also, reactive antibodies to SARS-CoV-2 S and N proteins are present in unexposed individuals, with virus neutralization activity reported from pre-pandemic pediatric samples (11, 12). It is postulated that this cross-reactive immunity may influence the nature and severity of COVID-19 symptoms upon infection and impact disease course (13) and may impact herd immunity.

Sensitive and accurate detection of virus-specific immune factors, such as antibodies, is imperative in order to measure rates of SARS-CoV-2 infections within communities with greater accuracy, to more fully define cross-reactive immunity in unexposed individuals, and to gain new understanding about the nature of effective versus potentially deleterious immune responses upon SARS-CoV-2 exposure. Antibody measurements are of particular importance, as pathogen-specific immunoglobulins are a known first line of defense upon exposure and can prevent new infections. Antibody titers are used to assess both likelihood of protection from re-infection and general vaccine efficacy (14). A variety of SARS-CoV-2 serological assays have been developed by multiple manufacturers and academic institutes and many are CE-marked and granted emergency use authorization (EUA) from the US Food and Drug Administration (FDA). Varieties include point-of-care rapid lateral flow assays, chemiluminescence immunoassays (CLIA), multi-plex bead/cell based-assays (15, 16), and enzyme-linked immunosorbent assays (ELISA) (17–20). These tests detect antibodies that primarily target the nucleocapsid protein (N) or the spike (S) protein of SARS-CoV-2, and specifically the Receptor Binding Domain (RBD) of spike which is an immunodominant surface protein targeted by neutralizing antibodies and a main target antigen for vaccine development (20–22). Some of these tests possess high sensitivity and specificity for detection of SARS-CoV-2 antibodies 14 days after diagnosis and/or symptom onset (23–26). However, others report negative results from individuals who are asymptomatic, mildly symptomatic, or symptomatic for less than 14 days, even when SARS-CoV-2 infection is confirmed (19, 27, 28); whether such individuals possess antibodies below the limit of the detection of the particular test used or lack these antibodies altogether is unresolved.

To enable detection of low levels of SARS-CoV-2-reactive antibodies, we modified the standard ELISA procedure, particularly the plate washing method, to improve sensitivity. Our protocol (the ‘BU ELISA’) allows clear SARS-CoV-2-reactive antibody signal resolution at sample dilutions as low as 1:5. Using this protocol we measured the levels of SARS-CoV-2-reactive IgM, IgG, and IgA from plasma or serum from three groups of individuals: (1) 71 subjects that varied by age, HIV infection, and systemic lupus erythematosus (SLE) disease status with all samples collected before November 8^th^, 2019 (‘pre-pandemic’); (2) 20 subjects hospitalized with COVID-19 (‘Acute’) (3) nine subjects with samples collected two-seven months after confirmed SARS-CoV-2 infection (‘Convalescent’). In addition, the performance of the BU ELISA, Antagen’s IgM IgG Lateral Flow Device (LFD) test and the Abbott IgG chemiluminescent microparticle immunoassay (CMIA) were directly compared from samples from the three subject groups.

## Material and Methods

### Participants

Pre-Pandemics: Samples were collected for unrelated studies prior to December 2019; these subjects included samples from a HIV and Aging cohort previously described in detail (29) and also from a geriatric cohort of subjects all over 60 years old with the following exclusion criteria: HIV, active hepatitis B or C, or any recent active infection within the past six months, diagnosis with an autoimmune disease, or treatment with any type of immunomodulatory therapy within 12 months. All pre-pandemic samples were collected before November 8^th^, 2019. Acute COVID-19: De-identified samples from hospitalized patients at Boston Medical Center with confirmed PCR positivity for SARS-CoV-2 comprise this group, with 10 subjects with positive and 10 with negative serology tests determined by the Abbott CMIA EUA approved assay. Samples were collected at various time points after onset of symptoms, (range 3-40 days); all samples were collected during the spring and summer of 2020, and all subjects in this group survived and were ultimately discharged from the hospital. Eighty percent of this group was comprised of males and 60% identified as black. SARS-CoV-2 Infected Convalescent: Subjects were recruited by contacting individuals who had been confirmed to have SARS-CoV-2 infection through their exposure at a biomedical conference in March 2020. None of the individuals were hospitalized. Samples were collected 2-7 months after a positive SARS-CoV-2 PCR test. The average age of this group was 52, with 22% male and 33% identified as black. All samples collected and/or used in this study with proper IRB approvals from the Boston University Institutional Review Board.

### The BU ELISA Protocol

Antibodies reactive to all four eCoV and SARS-CoV-2 RBD or N were assayed from sera or plasma as described in accompanying SOP (**Supplemental Figure 1**). Briefly, wells of 96-well plates (Pierce 96-Well Polystyrene Plates; cat#15041, Thermo Fisher Scientific) were coated with 50µl/well of a 2µg/ml solution of each respective protein in sterile PBS (Gibco) or with PBS only for 1 hour at room temperature. Coating solution was removed manually by a swift flick of the plates into a biohazard waste container. Next, 200µl per well of sterile PBS was added with a multichannel pipettor and liquid was removed *via* swift flick and the plate was banged on absorbent paper towels to remove residual liquid; this washing procedure was performed three times. Next, 200µl of casein blocking buffer (Thermo Fisher Scientific, cat#37528) was added to wells at room temperature for 1 hour. Next, plates were washed three times as previously described. Subject samples and monoclonal SARS-CoV-2 RBD reactive antibodies (IgG, clone CR3022, gift from the Ragon Institute; IgA, clone CR3022, Absolute Antibodies; IgM, clone BIB116, Creative Diagnostics) were diluted in Thermo Fisher casein blocking buffer, and 50µl of each were added to the plates for 1 hour at room temperature, with dilution buffer only added to blank wells. After incubation, samples were removed by a swift flick into a biohazard waste container. The plates were again washed three times with PBS containing 0.05% Tween 20 (PBST) and banged on absorbent paper towels, and immediately anti-human horseradish peroxidase (HRP)-conjugated secondary antibodies for IgG (cat#A18817, Thermo Fisher, 1:2000), IgM (cat#A18841, Thermo Fisher, 1:8000), and IgA (Jackson Immunoresearch, cat#109-035-011, 1:2000) diluted in casein blocking buffer were added to the plates at 50µl per well for 30 minutes at room temperature. Next, plates were washed four times with 0.05% PBST as described, and 50µl per well of 3,3’,5,5’-Tetramethylbenzidine (TMB)-ELISA substrate solution (Thermo Fisher Scientific, cat# 34029) was added and incubation occurred in the dark until a visible color difference between the well with the seventh dilution (1.37ng/ml) of recombinant antibody and the diluent only ‘zero’ well appeared, this time ranged from ~8-30 minutes. The reaction was stopped by adding 50µl of stop solution for TMB (Thermo Fisher Scientific, cat#N600) and the optical density was measured 450 nm (OD 450nm) on a SpectraMax190 Microplate Reader (Molecular Devices). Seven-point sample dilution curves were run in uncoated wells and paired antigen coated wells (SARS-CoV-2 RBD and N). An example of a plate map shown in **Supplemental Figure 1**. Samples were not run in a blinded manner as OD measurements are determined *via* a plate reader. To ensure accurate determination of antibody levels between subject groups, samples from pre-pandemic, acute COVID-19, and SARS-CoV-2 infected survivors were routinely run in mixed batches on ELISA plates and/or during experimental runs.

### Antigens

SARS-CoV-2 RBD was a gift from the Schmidt lab at the Ragon Institute and was expressed and purified as previously described (30). SARS-CoV-2 N (Cat# 40588-V08B) and S (Cat# 40591-V08H), NL63 N (Cat# 40641-V07E), 229E N (Cat# 40640-V07E), OC43 N (Cat# 40643-V07E) and HKU1 N (Cat# 40641-V07E) was purchased from Sino Biological. Histidine-tagged NL63 and HKU1 RBD sequences were inserted into plasmid vector VRc (gift from the Schmidt lab at the Ragon Institute) and was expressed in 293 Freestyle cells (293F, ThermoFisher) and purified on Ni-NTA resin as previously described (31).

### Determination of Arbitrary Units

Data were analyzed using GraphPad Prism 8. Arbitrary units (AU) on a ng/ml scale were calculated from the optical density (OD) values according to standard curves generated by known amounts of monoclonal anti-SARS-CoV-2 RBD IgG, IgM, or IgA. The OD values of blank (diluent only) wells with the same coat and secondary detection antibody were averaged and subtracted from the OD values of each respective sample well and then the ODs were logarithmically transformed. Next, a non-linear regression of the sigmoidal standard curve was used to extrapolate a “concentration” for the patient samples, which was then inverse log transformed and multiplied by the respective dilution factor. AU values for each sample were chosen from the linear portion of the dilution curve for the antigen coated wells, and the paired buffer only coat value was subtracted to determine the net AU amount.

### Determination of the Presence Versus Absence of SARS-CoV-2 Reactive Antibodies in Samples and of Arbitrary Unit Values:

First, the average ODs of corresponding ‘blank’ wells (sample diluent only in buffer only coated or antigen coated) on a given plate was subtracted from all wells with samples. ODs for blank wells was consistently ~0.05 regardless of coat. Metric 1: Signal was considered positive from a given subject if (1) the OD values from the antigen coated wells was a minimum of 2.5x higher than that of the paired buffer coated well for at least two sample dilutions and (2) one antigen-coated well OD value was over 0.1, after the average OD values of the respective blank wells were subtracted.

### LFD Tests

Antagen’s DISCOVID IgM IgG LFD test was used to detect SARS-CoV-2 RBD specific IgM and IgG antibodies following manufacturer instructions. Briefly, 20µl of plasma or serum was added to the indicated sample port, immediately followed by provided diluent, and incubated at room temperature before reading at 45 minutes. The results were scored as positive or negative for IgM and IgG by two independent readers blinded to donor sample status.

### Abbot Serology Test

The SARS-CoV-2 IgG assay is a chemiluminescent microparticle immunoassay (CMIA) used for the detection of SARS-CoV-2 nucleocapsid protein-specific IgG in human samples. The assays were performed according to manufacturer’s protocol.

### Automated Washer

**Figure 1:**
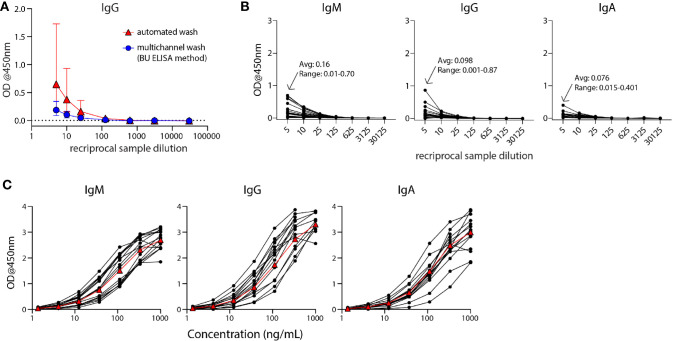
The modified ELISA (BU ELISA) protocol exhibits low background signal at high sample concentration and use of SARS-Cov-2 RBD-recombinant antibody standard curves allows for accurate sample quantification *via* accounting for OD drift between experimental runs. **(A)** Dilution curves of buffer only coated wells from five donor samples after using an automated plate washer or the BU ELISA method of multichannel plate washing. Experiment was performed once. **(B)** Representative dilution curves of buffer only coated wells from 30 subjects, average and range of 1:5 sample dilution for each isotype from all subjects; IgM, IgG, and IgA were detected in individual assays. **(C)** Representative IgM, IgG, and IgA standard curves from 15 different experimental runs are shown. The average of all runs shown as red triangles.

Plates were washed with Molecular Devices SkanWasher 400 microplate washer with three rounds of aspiration and wash with a final aspiration step for each run. This protocol was run twice after the coating, blocking, and sample incubation steps and three times after the addition of the secondary detection antibody step in the experiment shown in **Figure 1A**. Plates were rotated 180° between each run. Residual wash buffer was left in the plates (plates were not blotted post-wash) to mimic a fully automated system.

## Results

### A Modified ELISA Protocol Demonstrates Low Noise From High Concentration Human Serum and Plasma Samples

The Enzyme-Linked Immunosorbent Assay (ELISA) is a commonly used method for the measurement of analytes in a suspension sample. While low cost and easy to adapt in most lab settings, a limitation of this platform is high background from some biological samples at low sample dilutions. Specifically, optical densities (ODs) from sample dilutions lower than 1:100 is often sizeable and can mask the analyte of interest. This issue is particularly germane to serologic testing for SARS-CoV-2, as antibodies that are cross-reactive in unexposed individuals, newly generated in asymptomatic and/or recent infections, induced from an encounter with low viral dose, or waned post convalescence may be missed because levels are below the limit of detection of current assays. To address this issue, we have developed an ELISA protocol with unique steps to reduce non-specific signal at low sample dilutions. One change is the plate washing procedure, which is performed manually by an operator using a multichannel pipettor and includes agitation and soaking steps with repeated complete removal of residual fluid as described (**Supplemental Figure 1**). ELISAs were performed that compared buffer coated well OD values of five human plasma samples with plates washed with our method or an automated plate washer and the total levels of non-specifically bound IgG was determined. The manual washing procedure resulted in a notably lower average and range of ODs at 1:5, 1:10, and 1:25 dilutions as compared with the automated washer (**Figure 1A**). This BU ELISA protocol was run on samples from a total of 71 pre-pandemic and 29 SARS-CoV-2-infected subjects (**Table 1**), with paired antigen coated and buffer coated wells for six or seven sample dilutions (**Supplemental Figure 1**) for all subjects. The average ODs for buffer coated, 1:5 diluted sample loaded wells from all subject samples measured at this dilution were 0.16, 0.098, and 0.076 for IgM, IgG, and IgA respectively (**Figure 1B**). Given these low background OD values and the results from the wash method comparison, it’s possible that details of our protocol other than the washing method may contribute to these low background ODs, such as the type of plates, the blocking buffer/sample diluent used, and the number and placement of washing steps (*Methods* and **Supplemental Figure 1**). This buffer only coat ‘noise’ is remarkably consistent between multiple runs of a given sample (**Supplemental Figure 2**) and appears to be due to components within the sample, such as IgG and inflammatory factors (32) and not due to assay variability. Importantly, when ODs from uncoated wells with the same dilution of sample are not measured and properly subtracted, incorrect interpretation of results as positive can occur (33); therefore, the no coat values were subtracted from coated OD results for all results to determine the true antigen-specific signal. Also, detection antibodies were tested for specificity to confirm accuracy of isotype-specific readouts and the ability of our IgG detection reagent to measure all four IgG subclasses (**Supplemental Figure 3**).

**Table 1:** Cohort.

Cohort Characteristics	Age (average, range)	Sex, M (%)	Length of Symptoms (days average, range)
**Pre-pandemics** (n = 71)			
Healthy (n = 37)	**50 (21 - 96)**	**78**	**N/A**
HIV+ (n = 24)	**45 (22 - 79)**	**96**	**N/A**
SLE (n = 10)	**39 (23 - 69)**	**30**	**N/A**
**Acute** (n = 20)	**63 (48 - 84)**	**80**	**18 (3 - 40)^+^**
**Convalescent** (n = 9)	**53 (35 - 77)**	**22**	27 (0 - 61)*

+current length of symptoms at time of sample collection.

*total length of symptoms.

### Modification of ELISA Development Duration Based on Standard Curve Signal Detection Enables Accurate Comparison of Antibody Levels Between Experimental Runs by Minimizing Impact of OD Drift

During assay development we noted differences in OD values in different experimental runs even with strict adherence to all procedures and length of steps. Therefore, for all sample runs, we included a standard curve using recombinant monoclonal IgM, IgG, and IgA antibodies that recognize SARS-CoV-2 RBD for each of the respective isotype assays and stopped the development reaction when there was a visible difference between the seventh dilution (1.37ng/ml) of the standard and the ‘zero’ (sample diluent only) well. Addition of these standards and timing of development in this manner helped to ensure accurate calculation of the relative antibody levels (termed ‘Arbitrary Units’ (AU) on a ng/ml scale, calculated as described in Methods) between samples run on different days, plates, and/or by different operators. It is important to note that monoclonal antibody curves that recognize SARS-CoV-2 RBD were not used to quantify the exact number of antibodies in samples, as this cannot be calculated accurately due to the mixture of reactive immunoglobulins in human samples. Alternatively, we used SARS-CoV-2 RBD-specific monoclonal antibodies to determine AU levels all for RBD– and N– reactive antibodies; an approach used in other studies (34, 35) and provided reproducible and foreseeable values in this cohort. The OD values of the standard curves following this development procedure for the IgM, IgG, and IgA assays for 15 representative runs are shown (**Figure 1C**). The development time of these runs to complete visualization of the standard curve development ranged from ~8-30 minutes, demonstrating the need to adjust substrate incubation time per experimental run to maximize signal detection.

### SARS-CoV-2 RBD-Reactive Antibodies Were Detected at Low Levels in 44 Percent of Pre-Pandemic Samples

**Figure 2:**
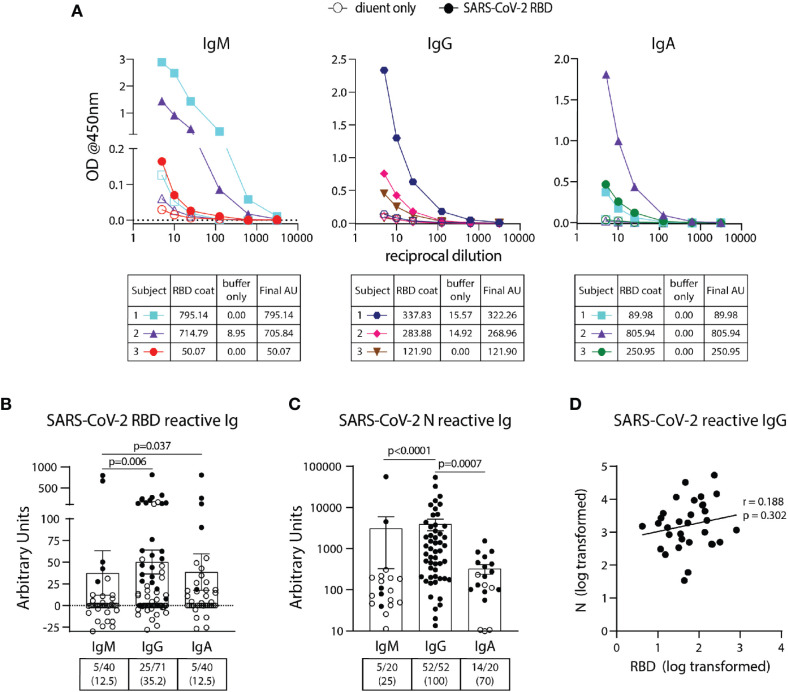
Detection and quantification of SARS-CoV-2 RBD- and N- reactive antibodies in pre-pandemic samples. **(A)** Representative dilution curves of seven pre-pandemic samples with SARS-CoV-2 RBD-reactive antibodies (three subjects per graph). Open and solid symbols represent buffer only coat and SARS-CoV-2 RBD coat, respectively. Arbitrary Units (AU) were calculated as described in Methods and shown beneath the respective isotype graph for diluent only and SARS-CoV-2 RBD coat. AUs for SARS-CoV-2 RBD **(B)** and N **(C)** reactive IgM, IgG, and IgA in pre-pandemic samples. Open and solid symbols represent negative and positive results, respectively, as determined by Metric 1. Enumeration of the positive samples for each isotype in the pre-pandemic cohort is shown beneath each graph with percentages of total in parentheses. **(D)** Correlation between AUs for IgG reactive to SARS-CoV-2 RBD and N (n=32). Values were log-transformed to obtain a parametric distribution. Statistical analyses were performed using an unpaired non-parametric Mann-Whitney t-test in **(B, C)** and Pearson’s correlation of normally distributed AU values for **(D)**.

SARS-CoV-2 RBD IgM, IgG, and IgA ELISA assays were performed on 40, 71, and 40 pre-pandemic samples, respectively (**Table 1**) using the BU ELISA protocol. The OD curves from the BU ELISA for both the buffer coat and SARS-CoV-2 RBD coated wells [after first subtracting the blank well(s) with paired coat] from seven representative pre-pandemic subjects with positive signal (three subjects per graph) for IgM, IgG, and/or IgA is shown (**Figure 2A**). There is clear RBD-specific signal with proportional loss of OD with sample dilution, providing evidence of true specific signal (**Figure 2A**). The calculated Arbitrary Units (AUs) from the buffer only and antigen coated wells from these curves is shown beneath each respective isotype graph. We defined a subject as positive for a given antibody readout as follows: the OD value from the RBD-coated well ≥ 2.5x the uncoated well from the paired sample dilution for at least two dilutions in the series and ≥ 0.1 for at least one dilution. Following this guideline, 31/71 of the unexposed individuals possessed reactive antibodies of at least one isotype to SARS-CoV-2 RBD, albeit all at very low levels in the circulation (~40 ng/ml) (**Figure 2B**). We compared the calculated AUs from IgG reactive to SARS-CoV-2 Spike (S) and RBD from 14 pre-pandemic subjects and found no significant correlation (**Supplemental Figure 4**). These results could be due to differences in portions and/or presentation of the RBD antigen in the different tests.

### All Pre-Pandemic Subjects Contained Circulating SARS-CoV-2 Nucleocapsid (N) IgG Antibodies With a Wide Range Of Levels Found Between Individuals

Using the BU ELISA protocol, we measured IgM, IgG, and IgA levels reactive with SARS-CoV-2 nucleocapsid protein (N) from 20, 52, and 20 subjects from our pre-pandemic cohort, respectively. Seven dilutions were run for all samples with and without N coated wells as with the SARS-CoV-2 RBD assays; sample dilution curves were generated, and positive results were determined using Metric 1 and AUs calculated. A wide range of pre-existing antibody levels were found; for example, the IgG levels varied by more than four fold, from roughly 0.0134 to 54 μg/ml (**Figure 2C**).

### No Correlation Between Levels of Cross-Reactive SARS-COV-2 Antibodies to RBD and N Antigens

We compared the levels of SARS-COV-2 RBD- and N- reactive antibodies between individual pre-pandemic subjects in our cohort and found no correlation between the two readouts (**Figure 2D**). This suggests these antibodies to different portions of SARS-CoV-2 are elicited during distinct immune responses.

### IgG Reactive With SARS-CoV-2 RBD and N in Pre-Pandemic Samples Correlate With Immunity to NL63 and Both NL63 and 229E eCoV Strains, Respectively

**Figure 3:**
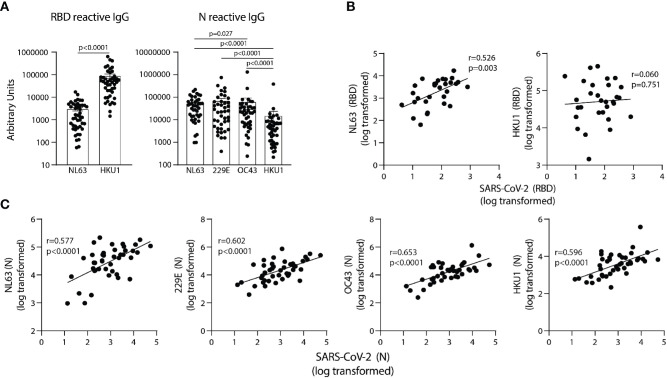
SARS-CoV-2 RBD and N reactive IgG in pre-pandemic samples track with IgG recognizing analogous proteins of eCoV strains. **(A)** AUs of IgG reactive to RBD of NL63 and HKU1 and N of all four eCoV strains (NL63, 2293, OC43, and HKU1). **(B)** Correlation between SARS-CoV-2 RBD IgG levels with NL63, HKU1 RBD IgG levels in individual subjects. **(C)** Correlation between SARS-CoV-2 N IgG and NL63, 229E, OC43, and HKU1 N IgG levels, n=30-42. Values were log-transformed to obtain a parametric distribution. Statistical analyses were performed using Pearson’s correlation of normally distributed log transformed AU values in **(B, C)** and an unpaired non-parametric Mann-Whitney t-test in **(A)**.

To determine if antibodies elicited by endemic coronavirus (eCoV) infections are linked to antibodies reactive to SARS-CoV-2 in unexposed subjects, IgG specific for NL63 and HKU1 RBD proteins, and IgG reactive with the N protein from all four eCoV strains in the circulation (NL63, HKU1, 229E and OC43) were measured. The levels of antibodies to eCoV RBD and N proteins in pre-pandemic samples showed general differences, with more IgG reactive to HKU1 than NL63 RBDs among the subjects and similar levels of IgG reactive with N proteins of the NL63, 229E, and OC43 strains, with lower levels reactive with the HKU1 N (**Figure 3A**). IgG reactive with SARS-CoV-2 RBD significantly correlated with NL63 and not HKU1 RBD-specific IgG (**Figure 3B**) and IgG reactive with SARS-CoV-2 N correlated with N-specific IgG for all four eCoV strains (**Figure 3C**). Taken together, these results suggest previous circulating endemic coronavirus infections elicit SARS-CoV-2 cross-reactive antibodies.

### HIV or SLE Disease Status Does Not Impact SARS-CoV-2 Reactive RBD and N Antibody Levels in Unexposed Individuals

**Figure 4:**
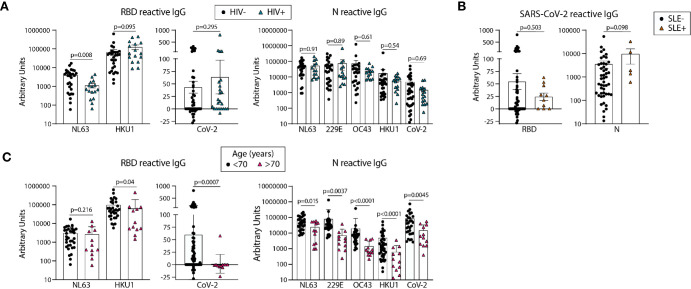
Older age is associated with lower circulating antibodies reactive with SARS-CoV-2 and eCoV RBD and N antigens. Quantification of IgG reactive to RBD of NL63, HKU1, and SARS-CoV-2 and N of NL63, 229E, OC43, HKU1, and CoV-2 in pre-pandemic samples regrouped based on HIV **(A)** or SLE **(B)** disease status or age **(C)**. Statistical analyses were performed using an unpaired non-parametric Mann-Whitney t-test.

We next compared the levels of eCoV and/or SARS-CoV-2 reactive antibodies in our pre-pandemic cohort with the subjects re-classified by HIV and SLE status. We found lower levels of NL63 RBD-reactive IgG in HIV+ as compared to uninfected subjects (**Figure 4A**); however, there were no other significant differences found between the antibody levels reactive to the RBD or N proteins, for either the eCoV strains or SARS-CoV-2, between groups classified *via* HIV or SLE status (**Figures 4A, B**).

### Unexposed Individuals Over 70 Years Old Have Significantly Lower Levels of SARS-CoV-2 RBD and N Reactive IgG Than Younger Counterparts

We next re-categorized our pre-pandemic cohort into two groups by age, <70yo (n=29-59) and >70 yo (n=12). All eCoV and SARS-CoV-2 reactive IgG levels measured were lower in the >70yo group, with high significance for SARS-CoV-2 RBD (p=.0007) and N (p=.0045). It should be noted that comparisons of younger (<35yo) and middle aged (40-65yo) groups did not yield notable differences (data not shown). Taken together, these results suggest that age may impact the magnitude of eCoV and SARS-CoV-2 cross-reactive antibody immunity more than a chronic viral infection (HIV) or an autoimmune disease (SLE).

### Comparison of SARS-COV-2 Specific RBD and N Antibody Levels Between Hospitalized COVID-19 Subjects With Acute Disease and Convalescent Survivors of Infection

**Figure 5:**
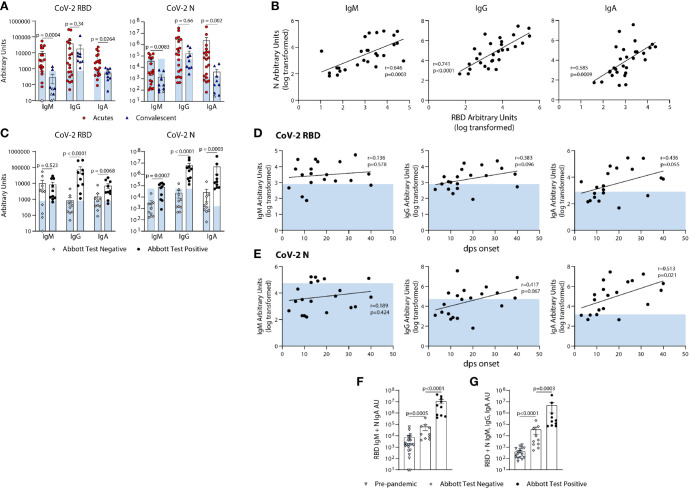
Quantification of the relative levels of IgM, IgG, and IgA-reactive SARS-CoV-2-RBD and N antibodies from acute and convalescent SARS-CoV-2 infected subjects. **(A)** Arbitrary Units (AUs) of SARS-CoV-2 RBD and N reactive IgM, IgG, and IgA of acute and convalescent subjects. Open and solid symbols represent negative and positive results, respectively, as determined by our Metric 1 described in Methods. **(B)** Correlation between SARS-CoV-2 RBD and N IgM, IgG, and IgA log transformed AUs. Values were log-transformed to obtain a parametric distribution. **(C)** Quantification of SARS-CoV-2 RBD and N reactive IgM, IgG, and IgA of acute subjects regrouped based on results from Abbott’s SARS-CoV-2 IgG CMIA. Correlation between SARS-CoV-2 RBD **(D)** and N **(E)** IgM, IgG, and IgA AUs (log transformed) with the number of days post symptom (dps) onset at time of sample collection for acute subjects. Quantification of SARS-CoV-2 RBD reactive IgM and N reactive IgA **(F)** and RBD & N reactive for IgM, IgG, and IgA **(G)** for pre-pandemics (n = 19) and Acutes re-classified based on Abbott test results. Light blue bars depict AU range of pre-pandemics for each respective antigen and isotype. Statistical analyses were performed using an unpaired non-parametric Mann-Whitney t-test in **(A, C, F, G)** and Pearson’s correlation of normally distributed log transformed AU values in **(B, D, E)** dps, days post symptom.

We next used the BU ELISA to measure the IgM, IgG, and IgA RBD- and N-specific antibodies from individuals at different times and magnitudes of severity after SARS-CoV-2 infection. Of the 20 COVID-19 hospitalized subjects (Acutes), 10 scored negative and 10 positive on the EUA approved Abbott SARS-CoV-2 N-specific IgG CMIA. RBD- and N- specific IgM and IgA was higher in all Acutes as compared with the Convalescent subjects, but IgG levels were similar, suggesting waning of IgM and IgA over time or reduced induction of these isotypes in subjects that do not require hospitalization (**Figure 5A**). Also, RBD- and N- specific IgM, IgG, and IgA levels significantly correlate among all COVID-19 subjects in the study (n=29) (**Figure 5B**).

### Comparison of RBD- and N-Specific Antibody Levels Measured by the BU ELISA From Hospitalized, Acute COVID-19 Subjects With Positive Versus Negative Abbott Test Results

Next, we compared the RBD- and N- SARS-CoV-2 specific antibody levels detected from the BU ELISA between Acute subjects with negative or positive Abbott IgG test results. The BU ELISA detected SARS-CoV-2 reactive antibodies from all samples, with all the Abbot test positive subjects with AU values above the pre-pandemic range for RBD- and N- specific IgG (**Figure 5C**). The AU values for six Abbott test negative subjects were above the pre-pandemic range for RBD-specific IgM and for N-specific IgA, and two Abbott test negative subjects have RBD-specific IgG above the pre-pandemic range (**Figure 5C**), Taken together, these iresults show clear serological evidence of infection in many of these Abbot test- subjects.

### Evidence of Diversity of Adaptive B Cell Response Induction Among Acutely Infected, Hospitalized COVID-19+ Subjects

Next, we compared the levels of RBD- and N- reactive IgM, IgG and IgA from our cohort of acute COVID-19 subjects, and found general trends showing higher antibody levels with more days of symptoms, with N-specific IgA significantly correlating with symptom length; however, some subjects have pre-pandemic levels of RBD- and/or N- reactive IgG, even after as long as 40 days of symptoms (**Figures 5D, E**).

### Combinational Analysis of Readouts by the BU ELISA Reveal SARS-CoV-2 Reactive Antibody Levels Are Significantly Higher in Acutely Infected COVID-19+ Subjects With Negative Abbott Test Results Than Pre-Pandemics

Next, we compared the combined AU values of both RBD- reactive IgM and N- reactive IgA and all six readouts performed and found significant differences between the pre-pandemic and acutely infected Abbott test negative groups, as well as between acutely infected Abbott test negative versus positive groups (**Figures 5F, G**). These results indicate that multi-parameter detection, comprised of multiple isotypes and antigen reactivities using a sensitive serology test could improve serologic diagnostics of SARS-CoV-2 infection.

### Three-Way Comparison of the BU ELISA With the Antagen LFD and Abbott CMIA Tests Reveal 9 of 10 Abbott Test Negative COVID-19 Subjects Exhibit SARS-CoV-2 Specific Antibody Levels Above All Pre-Pandemic Samples for at Least One Readout by the BU ELISA

The AU values from the 10 pre-pandemic subjects with the highest AU values for SARS-CoV-2 RBD and/or N-reactive IgG were directly compared with results from the Abbott CMIA assay and a lateral flow rapid test (Antagen Pharmaceuticals). The Abbott CMIA test measures IgG reactive to SARS-CoV-2 N, and the Antagen LFD test measures SARS-CoV-2 RBD-reactive IgG and IgM. Both commercial tests detected no SARS-CoV-2 reactive antibodies in the pre-pandemic samples, and correctly identified the infection status of 9/10 and 10/10 subjects within the convalescent group (LFD and Abbot tests, respectively) (**Table 2**). Among the acutely infected subjects, the 10 samples that scored positive for IgG the Abbott test were also positive for IgG the LFD test. Of the 10 acutely infected subjects that scored negative on the Abbott test, the LFD test successfully identified 5/10 subjects as SARS-COV-2 antibody positive via IgG and/or IgM (**Table 2**). Nine of 10 acute COVID-19 subjects that scored negative on the Abbott test have AU values above all pre-pandemics tested for at least one of the six BU ELISA readouts (all but Subject A4). There are some discrepancies between the different assays in detection of RBD- and N-reactive antibodies from the samples; this could be due to differences in the antigen preparations used, with varying expression vectors, impurities, tags, etc. These results underscore the importance of testing antigens from multiple sources and derivation methods, possible manipulation of antigens to minimize non-specific binding, and comprehensive assay validation to ensure maximal detection of true antigen specific signal.

**Table 2:** Comparison of SARS-CoV-2 reactive antibody results measured by the BU ELISA protocol, the EUA approved Abbott IgG chemiluminescent microparticle assay, and Antagen’s lateral flow rapid test.

Assay	ELISA						CMIA	LFD	
Antigen	RBD			N			N	RBD	
Isotype	IgM	IgG	IgA	IgM	IgG	IgA	IgG	IgM	IgG
**Pre-pandemic**									
P1	n.d.	811	n.d.	n.d.	1165	n.d.	**-**	**-**	**-**
P2	n.d.	322	n.d.	n.d.	500	n.d.	**-**	**-**	**-**
P3	n.d.	269	n.d.	n.d.	14579	n.d.	**-**	**-**	**-**
P4	n.d.	236	n.d.	n.d.	54025	n.d.	**-**	**-**	**-**
P5	n.d.	210	n.d.	n.d.	427	n.d.	**-**	**-**	**-**
P6	n.d.	201	n.d.	n.d.	434	n.d.	**-**	**-**	**-**
P7	n.d.	-5	n.d.	n.d.	18880	n.d.	**-**	**-**	**-**
P8	n.d.	112	n.d.	n.d.	12074	n.d.	**-**	**-**	**-**
P9	n.d.	76	n.d.	n.d.	9273	n.d.	**-**	**-**	**-**
P10	n.d.	125	n.d.	n.d.	6936	n.d.	**-**	**-**	**-**
**Acute**									
A1	54703	403	7861	933	15561	11067	**-**	**+**	**+**
A2	28388	171	2308	20037	1320	15811	**-**	**+**	**-**
A3	3091	306	383	3271	148738	221262	**-**	**+**	**-**
A4	74	158	201	158	4352	753	**-**	**+**	**-**
A5	3117	48	87	332	447	64	**-**	**-**	**-**
A6	7015	140	810	2354	462	2578	**-**	**-**	**-**
A7	127	609	1129	195	46225	1748	**-**	**-**	**-**
A8	460	4520	368	459	1255	1255	**-**	**+**	**-**
A9	0	1724	1034	193	1454	512	**-**	**-**	**-**
A10	3618	646	424	2377	5851	618	**-**	**-**	**-**
A11	1258	287496	12852	5871	2886248	178981	**+**	**-**	**+**
A12	3415	8722	3370	128747	401532	68030	**+**	**+**	**+**
A13	20404	39617	2736	15613	3805905	890383	**+**	**+**	**+**
A14	7068	1137	2290	167263	4543051	37356518	**+**	**+**	**+**
A15	1006	2243	836	63391	494137	118120	**+**	**+**	**+**
A16	20177	69285	16511	80754	28704356	392309	**+**	**+**	**+**
A17	29098	18804	6681	157527	127798	50048	**+**	**+**	**+**
A18	1560	49322	2869	118944	249225	68610	**+**	**-**	**+**
A19	678	7266	540	4922	1995094	7929190	**+**	**-**	**+**
A20	1341	268701	22853	803	17483216	218923	**+**	**-**	**+**
**Convalescent**									
C1	66	3186	311	269	37745	1658	**+**	**-**	**+**
C2	25	2630	1054	111	8759	34	**+**	**-**	**+**
C3	1377	120984	1150	5809	557571	20315	**+**	**+**	**+**
C4	57	9893	604	79	111654	13386	**+**	**+**	**+**
C5	11	5970	956	5049	82953	286	**+**	**+**	**+**
C6	25	6455	535	68	11921	29	**+**	**-**	**+**
C7	894	8918	198	1026	786367	640	**+**	**-**	**+**
C8	55	2897	255	271	9909	290	**+**	**-**	**-**
C9	0	1121	39	133	2584	55	**+**	**-**	**+**

BU ELISA AU values **(RBD)**: ≤ 795, 811, 806 (pre-pandemic range) : 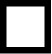 for IgM, IgG, IgA respectively ; 796, 812, 807 - 10,000 : 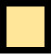 for IgM, IgG, IgA respectively ; 10,001 - 100,000 : 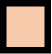 ; ≥100,001 : 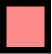

BU ELISA AU values **(N)**: ≤ 56230, 54025, 1544 (pre-pandemic range) : 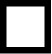 for IgM, IgG, IgA respectively ; 56231, 54026, 1545 - 100,000 : 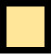 for IgM, IgG, IgA respectively ; 100,001 - 10^6^ : 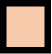 ; ≥10^6^ : 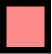

Negative as determined by Metric 1 : 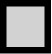

n.d. = not done

## Discussion

Accurate and sensitive measurement of virus-specific antibodies could complement diagnostic testing, provide information about the true prevalence of infection, provide insight into anti-viral immunity, and help assess vaccine responses. However, a lack of required sensitivity and specificity of many of the SARS-CoV-2 antibody tests available to date have led some to conclude that they have limited clinical utility in combating COVID-19 (36). Here, we present a modified ELISA protocol with exceptional sensitivity with high concentration samples that enables the detection of low levels of antigen-specific antibodies in human specimens.

The BU ELISA is straightforward, comprised of reagents that are readily available from commercial vendors and can easily be adapted for other applications and analytes. It should be noted that nine different operators have performed the BU ELISA to date and the reported signal:noise was always achieved with ease, even by individuals without prior experience running ELISAs. However, a limitation of this assay is that it is currently considerably lower in throughput compared to other serological platforms. This protocol requires an operator for the manual wash steps, limiting the number of plates that can be run compared to automated methods, however, there is potential for throughput increase if automated washers/ELISA systems can be adapted to more closely mimic this protocol. Specifically, we believe the manual wash procedure in this method minimizes cross-tip contamination, ensures thorough removal of wash buffer from the well, and prevents cross-well contamination. Application of these changes to an automated washer may improve sensitivity of higher-throughput serology test platforms. Another study limitation is the inclusion of only cross-sectional samples; in particular, it would be of high interest to measure SARS-CoV-2 antibody levels of the ten acute COVID subjects with negative Abbott test results both before infection and over time in the coming months to better understand the dynamics of the antibody response, e.g. to determine whether or not some antibody specificities/isotypes are never elicited in these infected individuals or if the quantities are low (still in the pre-pandemic range) but higher than pre-SARS-CoV-2 infection amounts. A third limitation of this work is the numbers of subjects included are not high enough to properly compare the specificity or sensitivity of the BU ELISA with commercial COVID-19 serology tests. Also, we were unable to measure RBD-specific IgG for two eCoV strains due to lack of availability (229E and OC43) which would be of interest given the lack of correlation between levels of reactive IgG to SARS-CoV-2 RBD with HKU1 RBD (**Figure 3B**).

Other important features of our approach include the inclusion of paired sample dilutions with buffer only coated wells to enable detection of true antigen-reactive signal and adjustment of the length of substrate incubation time based on standard curve development for OD standardization to enable direct comparison between samples on different plates. Quantification of relative antibody levels *via* Arbitrary Units (AU) or a similar method will be imperative for determining which convalescent samples have antibody levels sufficient for effective plasma transfer as well as other applications. However, while we believe this is a preferred approach for determining relative output values within all samples, it is critical to note that the unique dynamics of the panoply of antibodies of varying affinities and isotypes within a given specimen causes inherent confounding factors to serologic readouts. For example, a specimen with a high level of SARS-CoV-2 RBD reactive IgM antibodies could have a lower detected signal for IgG and IgA due to IgM’s pentameric conformation blocking many binding sites. Also, higher affinity antibody clones (IgG and IgA vs IgM, for example) may outcompete for binding sites of the coated antigen and thereby be detected more readily than others. We can account for this issue to some extent *via* measurement of all three major isotypes in all samples. Due to these unique dynamics of competition between immunoglobulins of different isotypes within an indirect ELISA assay, we did not use monoclonal antibodies for all antigen reactivities as standards; alternatively, we chose to employ one representative monoclonal antibody curve of a given isotype for AU calculations for all antigen-reactive IgM, IgG, and IgA quantification. This approach resulted in reproducible results enabling comparison of relative levels of antibodies specific for a several different antigen between all subjects with only one standard curve on each plate.

Here, we report links between antibody responses to endemic coronavirus (eCoV) strains and levels of cross-reactive antibodies with SARS-CoV-2 in unexposed individuals. Firstly, these results provide an important biological validation of the viral specificity of the OD values detected *via* use of the Ragon Institute SARS-CoV-2 RBD antigen and commercial N antigen (Sino Biologicals). Also, these findings support recent reports of SARS-CoV-2 T cell responses correlating with eCoV T cell memory in pre-pandemic samples. Importantly, hospitalized COVID-19 subjects with recent eCoV infections were significantly less likely to require ICU admission or succumb to the disease (37), and blood samples from virally unexposed children were found to possess neutralization activity against SARS-CoV-2 (11). These results collectively implicate past eCoV infections with protection from severe outcomes to COVID-19 due to cross-reactive immunity, including antibodies. Although all of the pre-pandemic subjects screened for SARS-CoV-2 N-reactive IgG scored positive in our assay, we found widely varying quantities (> four log differences) between subjects, an agreement with another study (12) and parallel reports of N-reactive T cell immunity in unexposed subjects (7–10). The magnitude of SARS-CoV-2 N reactive IgG in our pre-pandemic cohort did not track with HIV infection, SLE, or with age among subjects under 70 years old (**Figure 4**) but did correlate strongly with eCoV N IgG levels (**Figure 3**). These results strongly suggest that individuals with more recent eCoV infections and/or more robust eCoV immune responses could have some immune protection against COVID-19 *via* cross-reactive antibodies, and this may partially explain the profound diversity of outcomes that occur upon SARS-COV-2 infection. Screening for this cross-reactive immunity may provide new insight into an individual’s risk of poor infection outcomes.

44 percent of the pre-pandemic subjects possessed antibodies of at least one isotype that bind to SARS-CoV-2 RBD, albeit at very low levels. These results are in contrast with the conclusions of other reports, which state that RBD-reactive antibodies are not detected in unexposed individuals (17, 20). However, in these studies the assays were run at higher sample dilutions and therefore low signal may have been missed or misinterpreted as noise. While these RBD-reactive antibody levels are low in the blood, it is possible that they are present in higher concentrations in other sites, such as the mucosa. Also, as antibodies to RBD are associated with virus neutralization both *in vitro* and in animal models (18, 38–40), performing detailed functional analyses of plasma samples from pre-pandemic samples with RBD-reactive antibodies is an important next step. Preliminary experiments from our group indicated that neutralization activity was not present in four subjects (data not shown) but future experiments are needed to more thoroughly address this question.

Unexposed individuals over 70 years of age possessed lower levels of both eCoV and cross-reactive SARS-CoV-2 antibodies (**Figure 4C**). A study comparing pre-existing SARS-CoV-2 reactive T cell immunity in different age groups found that levels were lower with older age (41). As individuals over 70 are more likely to present with serious COVID-19 complications (42–45) future research investigating connections between age, eCoV and SARS-CoV-2 reactive immunity, and vulnerability to severe COVID-19 is warranted.

Direct comparison of our ELISA protocol with two commercially available serological assays for SARS-CoV-2, Antagen’s LFD test and Abbott’s CMIA IgG assay yielded interesting results. Both the LFD and CMIA performed well in identification of the convalescent subjects *via* detection of SARS-CoV-2 IgG to RBD (Antagen test) and N (Abbott test), and the BU ELISA detected signal from many pre-pandemic samples which all scored negative in the Antagen and Abbott tests (**Table 2**). However, these commercial tests are specifically designed to detect SARS-CoV-2 infection, unlike the BU ELISA which is measuring all SARS-CoV-2 reactive antibodies; therefore, it is possible the antigens have been modified in the commercial assays to minimize cross-reactive antibody detection, designated as noise in these tests. Interestingly, of the 10 acute subjects that scored negative on the Abbott test, five scored positive for IgM and/or IgG by the LFD test; also, of the six parameters measured by the BU ELISA, AU values were higher than all pre-pandemic samples for at least one readout for 9/10 subjects. Taken together, these results indicate multi-parameter detection of SARS-CoV-2 reactive antibodies with sensitive tests may improve use of serologic data for diagnostics.

The BU ELISA protocol enables the measurement of low levels of antigen-specific antibodies within high concentration human specimens. Use of this assay could provide new insight into viral transmission and help elucidate the nature of the virus-specific antibody response. Also, this protocol may aid diagnostics, measurement of vaccine responses and perhaps most importantly, help accurately determine a history of exposure to SARS-COV-2.

## Data Availability Statement

The raw data supporting the conclusions of this article will be made available by the authors, without undue reservation.

## Ethics Statement

The studies involving human participants were reviewed and approved by Boston University Institutional Review Board. Written informed consent for participation was not required for this study in accordance with the national legislation and the institutional requirements.

## Author Contributions

JS-C conceived of the experimental plan. RY and JS-C designed the approach. AC, RY, and JS-C designed experiments. RC, NL, KQ, PU, NB, GM, WG, IR, MS, and JS-C contributed to study subject specimen collection/use, provided reagents, and/or funding. RY, ES, RP, DS, EC, JS-C, AO, LB, FK, JB, AB, and YK performed experiments. RY, DS, EC, JB, SG, AB, GM, YK, and JS-C analyzed data. RY and JS-C wrote the manuscript. All authors contributed to the article and approved the submitted version.

## Funding

This work was supported by the Boston University's National Emerging Infectious Diseases Laboratories (NEIDL) Director's Fund, NIH grants R01AG06505-01 and R01AG058538-01, and sample collection was partially supported by the Providence/Boston Center for AIDS Research (CFAR).

## Conflict of Interest

WG was employed by Antagen Pharmaceuticals.

The remaining authors declare that the research was conducted in the absence of any commercial or financial relationships that could be construed as a potential conflict of interest.
