# Supplementary figures and images for: Novel ELISA Protocol Links Pre-Existing SARS-CoV-2 Reactive Antibodies With Endemic Coronavirus Immunity and Age and Reveals Improved Serologic Identification of Acute COVID-19 via Multi-Parameter Detection
Source: Front Immunol. 2021 Apr 9;12:614676. doi: 10.3389/fimmu.2021.614676 (PMC8062931; doi:10.3389/fimmu.2021.614676)

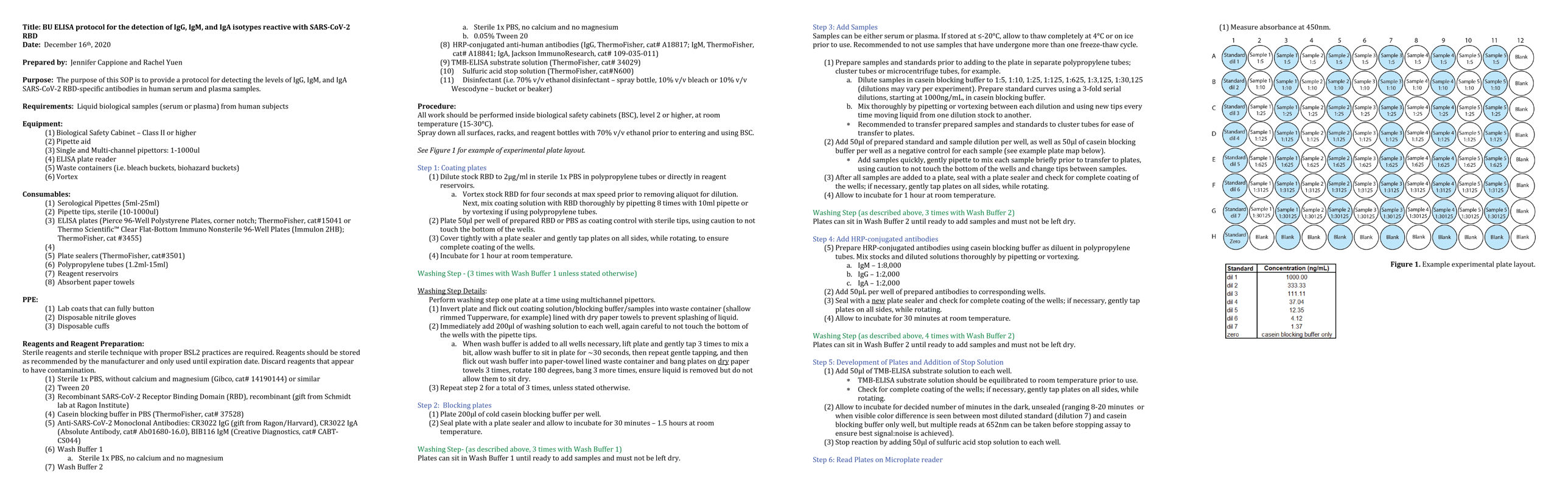

Supplement: Supplementary file 1 [file Image_1.jpeg]

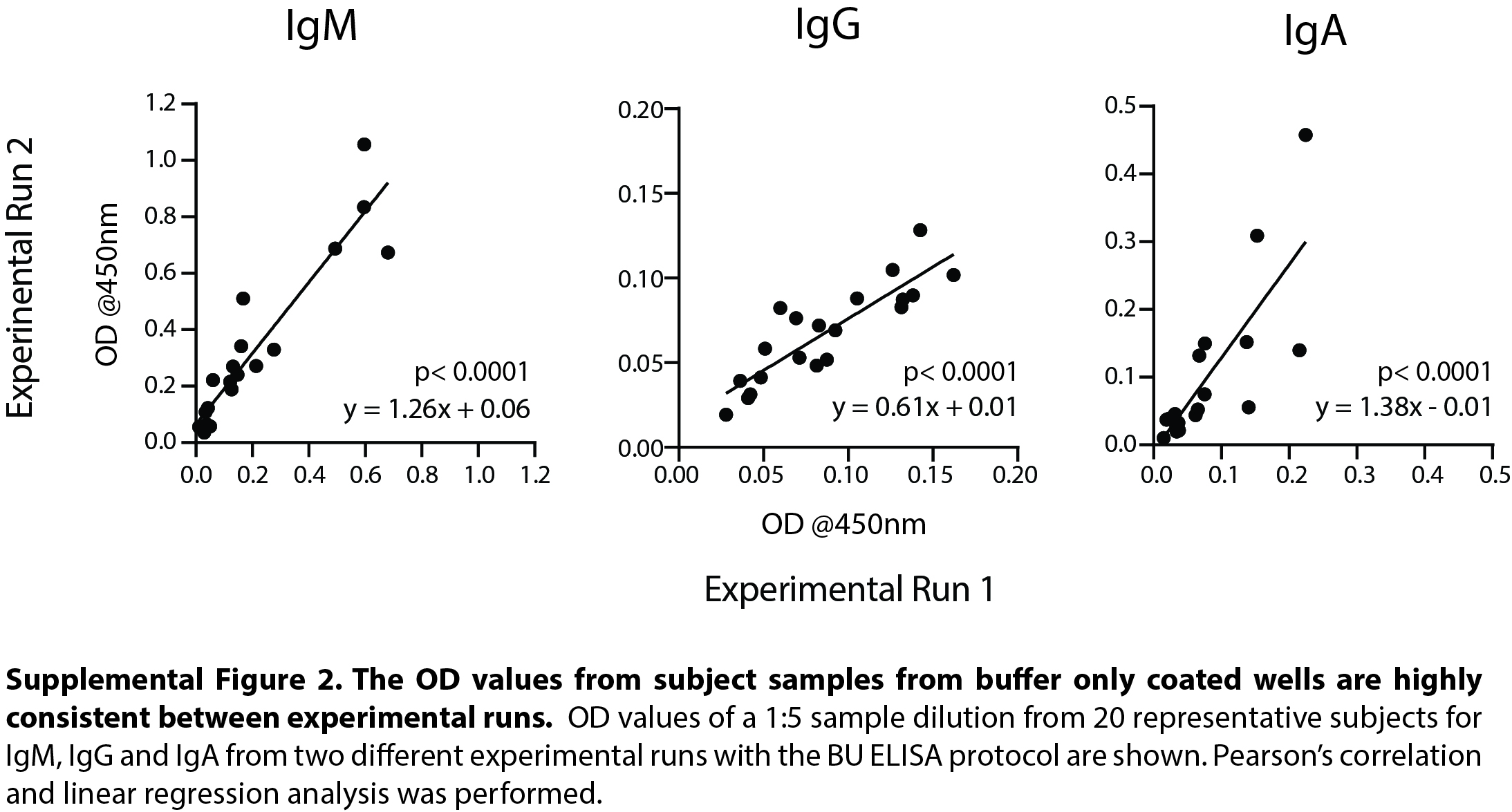

Supplement: Supplementary file 2 [file Image_2.jpeg]

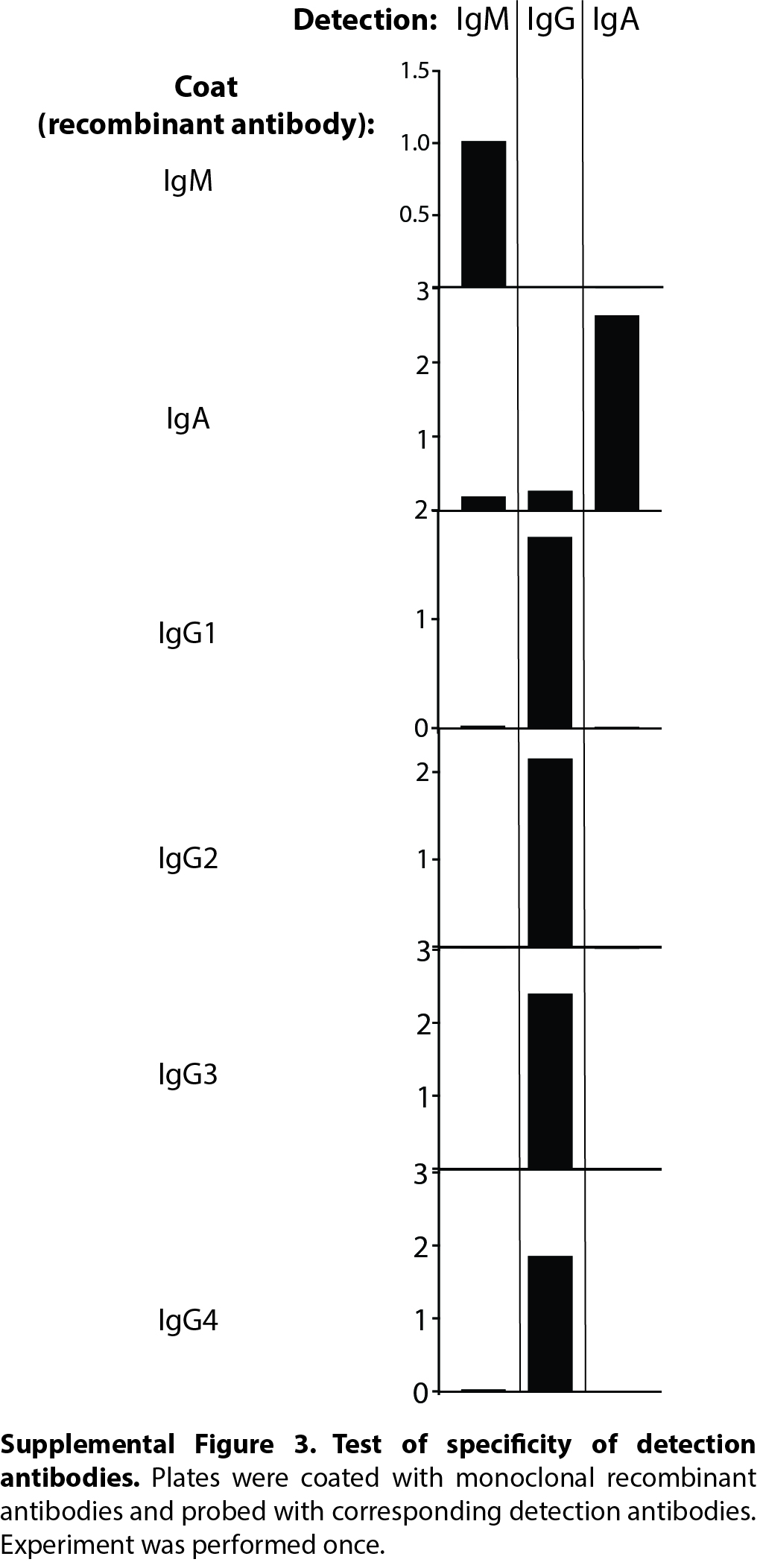

Supplement: Supplementary file 3 [file Image_3.jpeg]

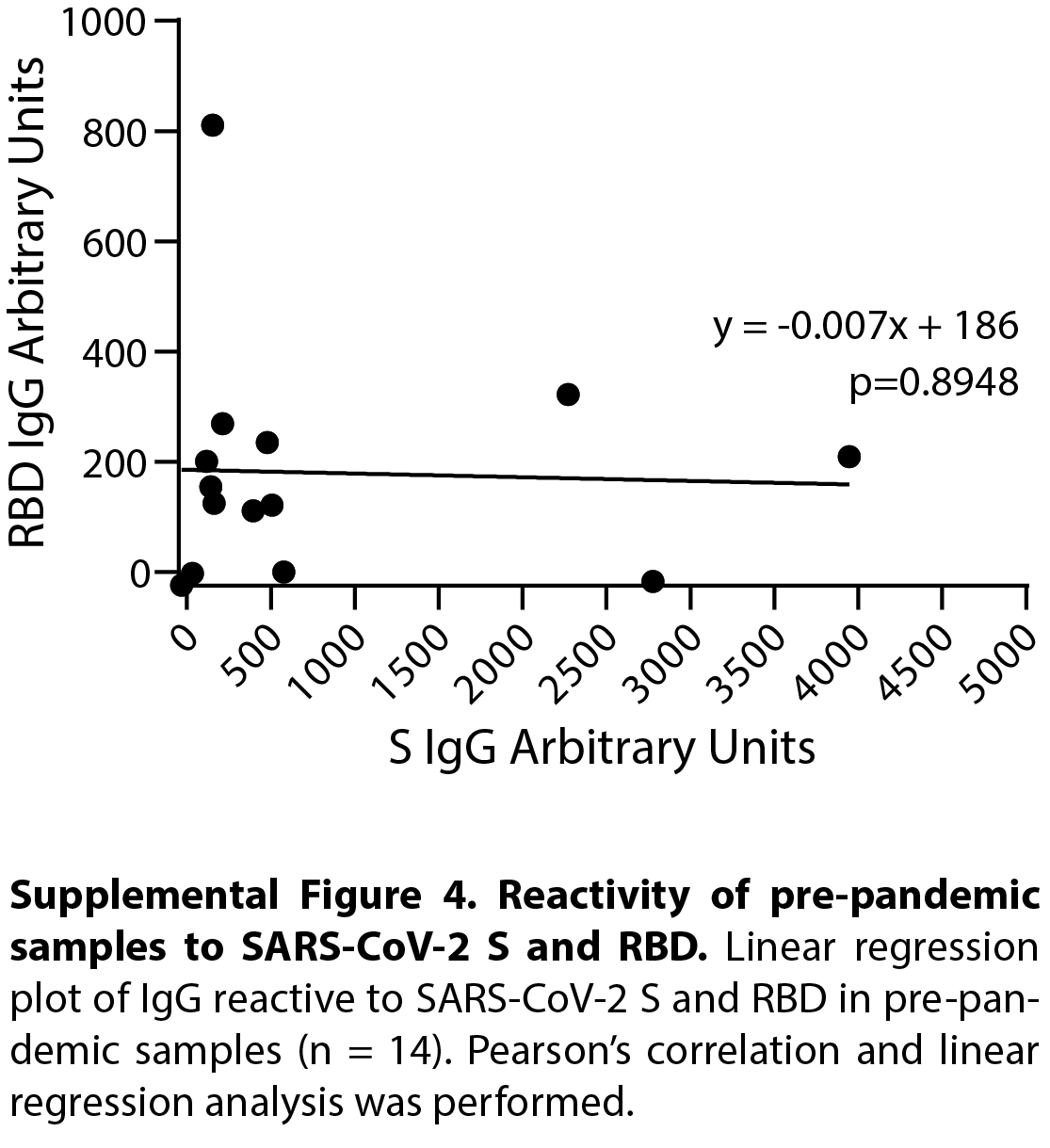

Supplement: Supplementary file 4 [file Image_4.jpeg]
